# Preoperative CT-derived thigh muscle quality as a predictor of postoperative outcomes after total knee arthroplasty in patients with hemophilia

**DOI:** 10.3389/fmed.2026.1877333

**Published:** 2026-08-11

**Authors:** Rui Feng, Jiaqi Yang, Yang Liu, Xiangang Luo, Fan Yang, Qunqun Chen

**Affiliations:** 1 The Third Clinical College of Guangzhou University of Chinese Medicine, Guangzhou, China; 2 The Third Affiliated Hospital of Guangzhou University of Chinese Medicine, Guangzhou, China

**Keywords:** computed tomography, hemophilia, haemophilic arthropathy, hounsfield units, muscle quality, total knee arthroplasty

## Abstract

**Background:**

Postoperative recovery after total knee arthroplasty (TKA) for haemophilic arthropathy varies substantially, and objective imaging markers for preoperative risk stratification remain limited. This study investigated whether computed tomography (CT)-derived thigh muscle quality and tissue composition are associated with 1-year outcomes after TKA in patients with hemophilia.

**Methods:**

This retrospective study included 67 patients with hemophilia who underwent primary unilateral TKA between January 2021 and March 2025. Preoperative CT was used to quantify thigh muscle volume, muscle quality, defined as mean muscle attenuation in Hounsfield units (HU), intermuscular adipose tissue, and subcutaneous adipose tissue. The excellent outcome was defined as both Knee Society Clinical Score (KSC) ≥70 and Knee Society Functional Score (KSF) ≥70 at 1 year. Multivariable linear regression, a prespecified five-variable logistic regression model, receiver operating characteristic (ROC) analysis, and inter-observer reproducibility assessment were performed. A secondary analysis compared CT-derived tissue parameters with age-, sex-, and body mass index-matched non-arthropathic controls.

**Results:**

CT-derived parameters showed excellent inter-observer reproducibility. Muscle quality was higher in the excellent-outcome group than in the non-excellent-outcome group (36.07 ± 4.08 vs. 30.04 ± 6.12 HU; *p* < 0.001). At 1 year, the excellent-outcome group had higher KSC (88.21±5.80 vs 84.55±6.55; p=0.038) and KSF (83.81 ± 5.46 vs. 63.00 ± 11.05; *p* < 0.001). Muscle quality was independently associated with postoperative SF-12 PCS (*p* = 0.006), KSC (*p* = 0.008), and KSF (*p* = 0.002). In logistic regression, muscle quality was independently associated with excellent outcome (OR = 1.30 per 1-HU increase; 95% CI, 1.11–1.50; *p* = 0.004). ROC analysis identified an exploratory cutoff of 31.4 HU (AUC = 0.829; 95% CI, 0.72–0.94), sensitivity of 80.0%, and specificity of 88.9%. Compared with matched controls, patients with haemophilic arthropathy had lower muscle volume and muscle quality but higher adipose tissue volumes.

**Conclusion:**

Preoperative CT-derived thigh muscle quality was independently associated with 1-year functional recovery after TKA in haemophilic arthropathy and may support preoperative risk stratification.

## Introduction

Hemophilia, a condition of blood coagulation resulting from a shortage of coagulation factors, primarily presents as bleeding of varied severity and locations (1). Coagulation factor deficiency (VIII or IX) is used to categorize patients with hemophilia (PWH) into categories A and B (2). Haemophilic arthropathy is a persistent and progressive outcome of repeated hemarthroses, resulting in structural joint deterioration, discomfort, deformity, and functional impairment, ultimately diminishing quality of life (3, 4). Total knee arthroplasty (TKA) is frequently required to relieve pain and restore function (5). Recovery trajectories and clinical outcomes vary significantly among individuals. However, evidence guiding optimal surgical timing and preoperative risk stratification remains limited. Identifying preoperative predictors of postoperative outcomes can inform patient selection and perioperative optimization to improve recovery.

Skeletal muscle health is increasingly being recognized as a determinant of perioperative resilience and post-arthroplasty function (6, 7). Importantly, “muscle status” in clinical practice reflects not only reduced muscle mass, but also impaired muscle quality, such as increased intramuscular fat infiltration, decreased muscle density, and the resulting limitations in strength and functional output (8). Computed tomography (CT) enables the simultaneous quantification of muscle volume, muscle attenuation in Hounsfield units (HU), and intermuscular adipose tissue (IMAT), providing a pragmatic imaging-based approach for preoperative assessment of muscle composition (9, 10). In osteoarthritis cohorts undergoing TKA, CT-derived quadriceps attenuation has been associated with postoperative physical activity and functional outcomes, and preoperative lower-extremity muscle composition has been associated with gait performance after TKA, supporting a biologically plausible link between muscle quality and recovery trajectories (11, 12). In haemophilic arthropathy, pain-related disuse, recurrent hemarthroses, and ongoing inflammation may act synergistically to accelerate lower-extremity muscle wasting and deteriorate the muscle quality (13, 14). Based on our clinical observations, patients with haemophilic knee arthropathy often demonstrate marked thigh muscle wasting and reduced functional reserve, yet the extent to which preoperative CT-derived thigh muscle quality and volume predict postoperative prognosis after TKA in patients with hemophilia remains to be established.

Therefore, this study aimed to investigate whether preoperative CT-derived thigh muscle quality is associated with 1-year postoperative outcomes after TKA in patients with haemophilic arthropathy. This imaging-based assessment may provide a useful basis for future studies aimed at improving preoperative risk stratification and predictive modeling of postoperative recovery in this population.

## Methods

### Patients

We retrospectively obtained and analyzed clinical information from patients with hemophilia (PWH) who underwent total knee arthroplasty (TKA) at the Orthopedic Center between January 2021 and March 2025. This study was approved by the Ethics Council of the Third Affiliated Hospital of Guangzhou University of Chinese Medicine (PJ-XS-20260205-003) and was registered in the Chinese Clinical Trial Registry (ChiCTR2600120082).

The inclusion criteria were as follows: (1) a confirmed diagnosis of hemophilia A/B; (2) knee joint abnormalities that fulfill the diagnostic standards of Class IV or V as per the Improved Arnold-Hilgartner classification (15); and (3) undergoing primary unilateral total knee arthroplasty at the Orthopedic Center.

**Figure 1:**
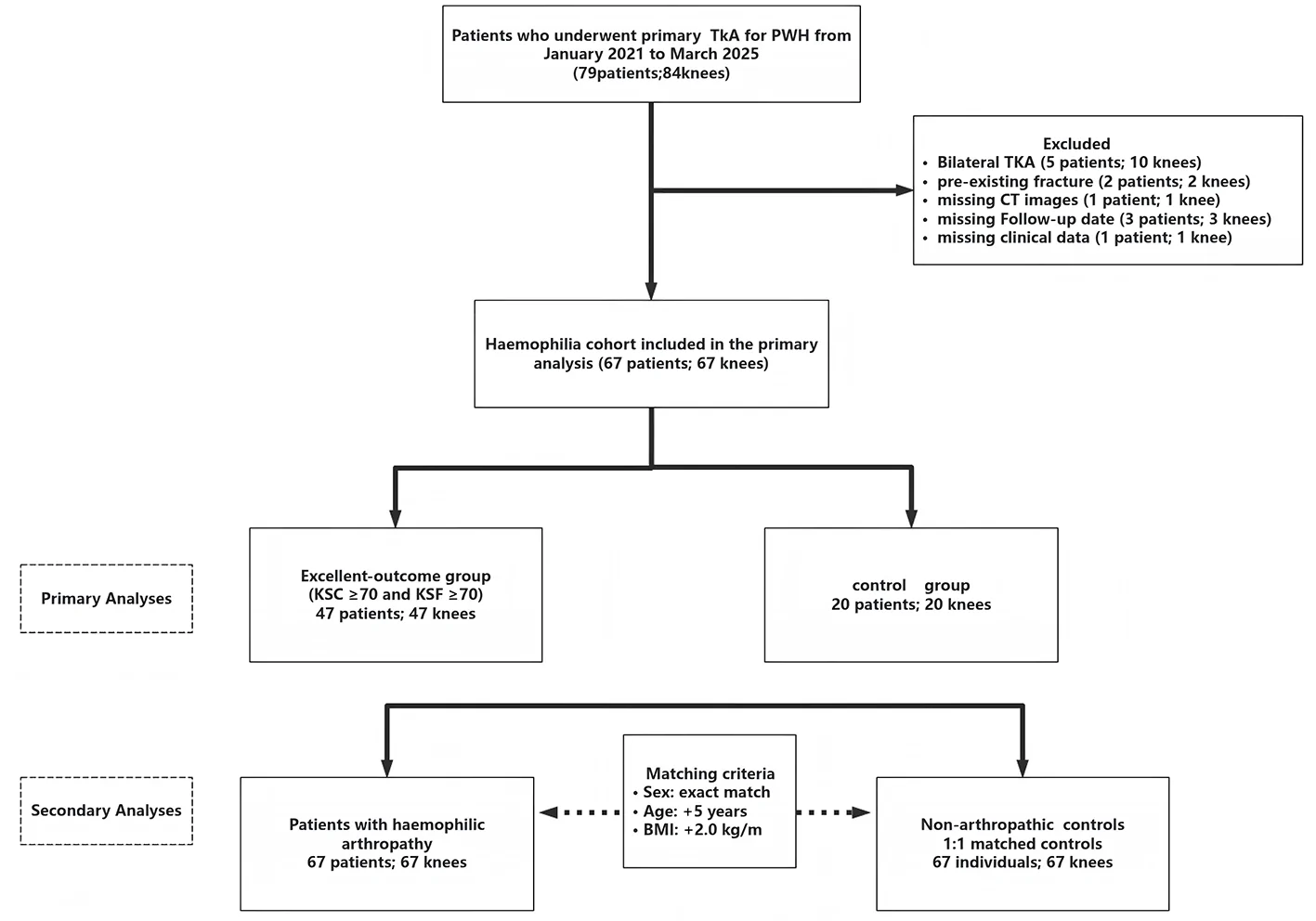
Flowchart of patient selection.

The exclusion criteria were as follows: (1) inadequate information, including demographic, imaging, and follow-up data; (2) bilateral total knee arthroplasty; (3) pre-existing fracture, amputation, or other major lower-extremity conditions (Figure 1).

For the secondary matched-control analysis, patients with haemophilic arthropathy were individually matched 1:1 to non-arthropathic controls using a prespecified matching scheme. Exact matching was required for sex, and age and body mass index (BMI) were matched within predefined calipers of ± 5 years and ±2.0 kg/m^2^, respectively. Controls were selected from individuals who underwent lower-extremity CT for reasons unrelated to knee arthropathy and had no radiographic evidence of knee osteoarthritis, inflammatory arthritis, previous knee surgery, fracture, or neuromuscular disease. To minimize information bias, the first author had no access to CT-derived measurements related to muscle quality (MQ), intermuscular adipose tissue (IMAT), or subcutaneous adipose tissue (SAT) during the matching step. The CT-based quantification of fat and muscle was incorporated into the final analytic dataset only after matching was finalized.

### CT imaging and measurement

CT scans were acquired using a Toshiba Aquilion CT scanner. For patients with more than one preoperative CT scan, the scan acquired closest to the index TKA was selected for quantitative analysis. All CT scans used in this study were obtained within 7 days before surgery. The scanning range was from 8 cm above to 5 cm below the femoral condylar line, with scanning parameters set to 120 kVp, 200 mA, and a 2.0 mm slice thickness. Images were reconstructed with a 512 × 512 matrix using a standard soft-tissue reconstruction kernel. For both hemophilia patients and non-arthropathic controls, tissue composition was quantified within the same predefined distal thigh region, extending from the femoral condylar line to 8 cm proximal to the femoral condyles.

**Figure 2:**
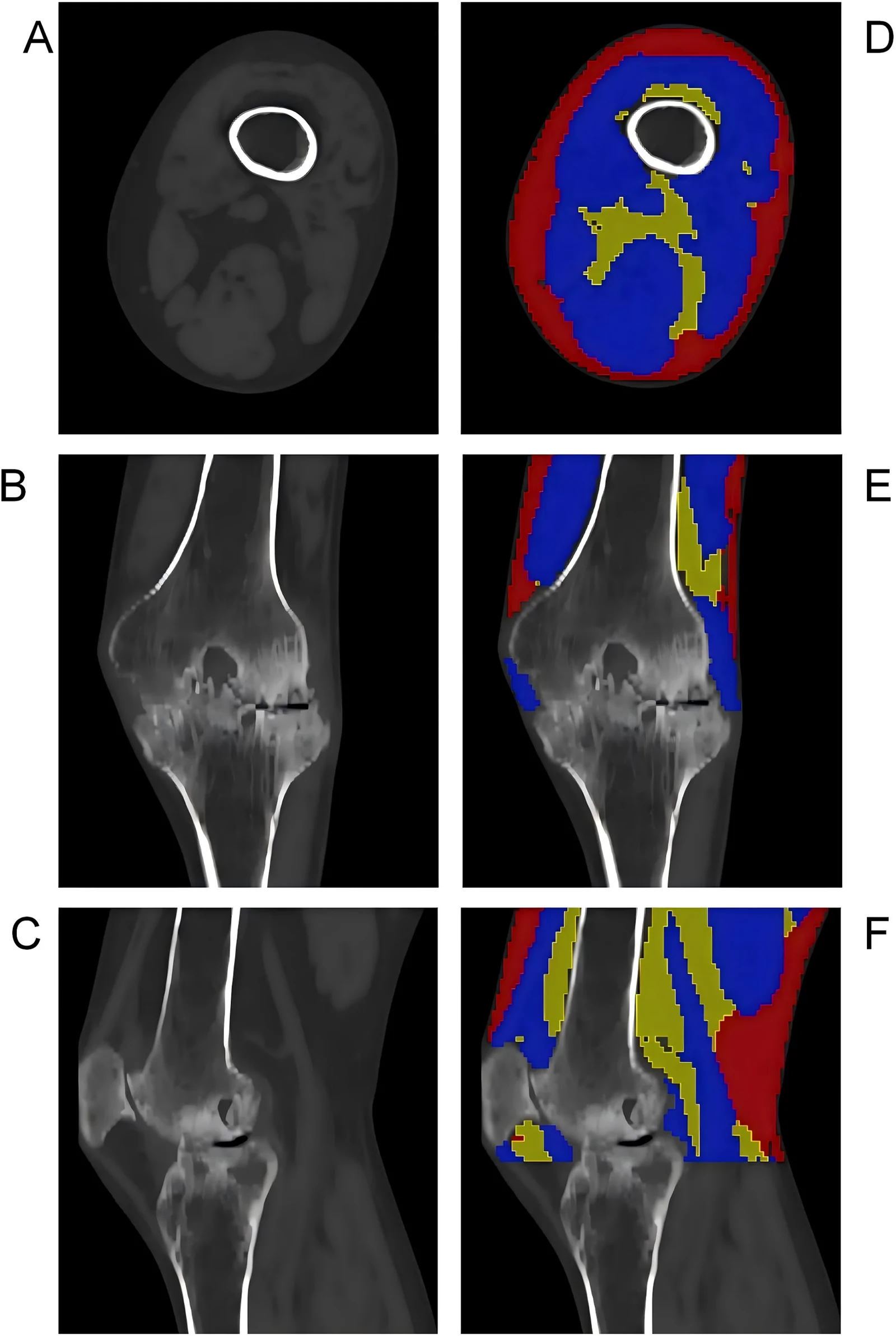
Representative CT-based segmentation of thigh muscle and adipose tissue compartments within the predefined distal thigh region in a patient with hemophilia. The region of interest extended from the femoral condylar line to 8 cm proximal to the femoral condyles. **(A–C)** show the original CT images in the axial, coronal, and sagittal planes, respectively. **(D–F)** show the corresponding segmentation masks after automatic segmentation and threshold-based tissue classification. Skeletal muscle is shown in blue, intermuscular adipose tissue (IMAT) in yellow, and subcutaneous adipose tissue (SAT) in red.

The TotalSegmentator was used for automatic segmentation of the femur and surrounding soft tissues (Dice coefficient: 0.943) (16). Tissue masks were subsequently classified using predefined HU ranges: skeletal muscle, −29 to 150 HU; adipose tissue, −190 to −30 HU (17, 18). TotalSegmentator is a deep learning model based on the nnU-Net (self-configuring 3D U-Net) architecture that is pretrained on large-scale CT imaging data to automatically identify and segment multiple anatomical structures of the human body (19). The model has been published and is open-source, demonstrating excellent generalization ability and clinical application potential (20). Following automated segmentation, two independent observers with experience in musculoskeletal imaging reviewed the segmentation masks for anatomical plausibility and performed corrections when necessary. To assess inter-observer reproducibility, a random subset of 30 CT scans was independently re-evaluated by the second observer. Inter-observer reliability was assessed for muscle quality, skeletal muscle volume, IMAT volume, and SAT volume using intraclass correlation coefficients (ICCs) with 95% confidence intervals. Both observers were blinded to clinical outcomes and group allocation during the reproducibility assessment. Representative segmentation results are shown in Figure 2.

Skeletal muscle volume, IMAT volume, and SAT volume were quantified separately using the voxel-counting method and expressed in cubic centimeters (cm^3^). Skeletal muscle volume was calculated from voxels classified as skeletal muscle within the predefined distal thigh region and did not include IMAT. IMAT was defined as adipose tissue located beneath the muscle fascia and between muscle groups. MQ was defined as mean skeletal muscle attenuation in HU and reflected compositional changes within the skeletal muscle compartment, including fatty infiltration (21, 22).

### Patient characteristics and postoperative outcomes

Baseline demographic and disease-related variables were collected, including sex, hemophilia type, body mass index (BMI), and Arnold-Hilgartner grade. Range of motion (ROM) and flexion contracture were assessed 1 d before surgery and at 1-year follow-up. Clinical outcomes were evaluated preoperatively and 1 year postoperatively using the Knee Society Score (KSS), Visual Analog Scale (VAS), and 12-item Short Form Health Survey (SF-12). The KSS comprises a clinical subscore (KSC) and a functional subscore (KSF), while the SF-12 provides the Physical Component Summary (PCS) and Mental Component Summary (MCS). Postoperative knee outcomes were categorized into an excellent outcome group and non-excellent-outcome group. Patients meeting both KSC ≥70 and KSF ≥70 were classified as the excellent outcome group, and those below either threshold were assigned to the non-excellent-outcome group.

### Statistical analyses

Statistical analyses were performed using DecisionLime (v1.1.1). For the primary analysis, PWH were stratified into excellent-outcome vs. non-excellent-outcome groups and compared using *t*-tests or Mann-Whitney U tests after Shapiro-Wilk normality assessment, and categorical variables were analyzed appropriately and summarized as n (%). Multivariable linear regression was used to evaluate associations between CT-derived body-composition metrics and postoperative KSC, KSF, and SF-12 PCS/MCS. Candidate covariates were selected *a priori* based on clinical relevance and prior literature rather than automated stepwise selection, and included age, BMI, preoperative flexion contracture, skeletal muscle volume, and muscle quality. Given the limited sample size, the multivariable logistic regression model was restricted to these five prespecified variables to reduce overfitting. Odds ratios (ORs) with 95% confidence intervals (CIs) were reported. Multicollinearity among candidate predictors was assessed using variance inflation factors (VIFs), with VIF values >5 considered indicative of potentially relevant multicollinearity. Receiver operating characteristic (ROC) curve analysis was used to determine the optimal cutoff value for excellent-outcome classification. Additional sensitivity analyses were performed to assess the robustness of the primary findings. IMAT volume was additionally included in the multivariable linear regression models, and an expanded logistic regression model was further adjusted for additional clinically relevant covariates. Subgroup analyses and interaction testing were performed to explore the consistency of the association across clinically relevant subgroups. Secondary analyses compared CT-derived muscle and adipose tissue parameters between patients with haemophilic arthropathy and 1:1 age-, sex-, and BMI-matched non-arthropathic controls using paired *t*-tests or Wilcoxon signed-rank tests. Inter-observer reproducibility of CT-derived parameters was evaluated using ICCs with 95% confidence intervals. A two-way random-effects model with absolute agreement was used. All tests were two-sided, and *p* < 0.05 was considered statistically significant.

## Results

### Measurement reproducibility

In the inter-observer reproducibility analysis, CT-derived parameters demonstrated excellent agreement between observers. The ICC was 0.94 (95% CI, 0.88–0.97) for MQ, 0.96 (95% CI, 0.93–0.98) for skeletal muscle volume, 0.91 (95% CI, 0.87–0.94) for IMAT volume, and 0.96 (95% CI, 0.91–0.98) for SAT volume.

### Primary analyses

Table 1 summarizes the baseline characteristics, CT-derived tissue composition, and postoperative outcomes stratified by outcome status. The excellent-outcome and non-excellent-outcome groups were comparable in demographics, hemophilia type, disease severity, BMI, and preoperative knee status (all *p* > 0.05).

**Table 1:** Demographic and clinical characteristics of patients with haemophilia.

Variable	Overall	Excellent outcome group	Non-excellent-outcome group	*p*-value
	*N* = 67	*N* = 47	*N* = 20	
Age, mean ± SD	38.66 ± 8.91	38.23 ± 8.46	39.65 ± 10.05	0.585
Sex, *n* (%)				—
Male	67 (100.0%)	47 (100.0%)	20 (100.0%)	
Female	0 (0.0%)	0 (0.0%)	0 (0.0%)	
Hemophilia severity, *n* (%)				0.434
Mild	0 (0.0%)	0 (0.0%)	0 (0.0%)	
Moderate	9 (13.4%)	5 (10.6%)	4 (20.0%)	
Severe	58 (86.6%)	42 (89.4%)	16 (80.0%)	
Hemophilia type, *n* (%)				0.342
A	60 (89.6%)	41 (87.2%)	19 (95.0%)	
B	7 (10.4%)	6 (12.8%)	1 (5.0%)	
Arnold-Hilgartner grade, *n* (%)				0.927
IV	24 (35.8%)	17 (36.2%)	7 (35.0%)	
V	43 (64.2%)	30 (63.8%)	13 (65.0%)	
BMI, mean ± SD	23.72 ± 3.52	23.79 ± 3.78	23.54 ± 2.91	0.769
Pre-FC, mean ± SD	15.94 ± 12.24	15.70 ± 11.73	16.50 ± 13.68	0.821
Pre-ROM, mean ± SD	49.28 ± 26.41	49.13 ± 27.05	49.65 ± 25.51	0.940
Pre-VAS, mean ± SD	2.97 ± 0.46	2.98 ± 0.44	2.95 ± 0.51	0.828
Skeletal muscle volume, cm^3^, mean ± SD	459.79 ± 165.23	464.44 ± 159.48	448.87 ± 181.88	0.742
MQ (HU), mean ± SD	34.27 ± 5.48	36.07 ± 4.08	30.04 ± 6.12	<0.001
IMAT volume, cm^3^, mean ± SD	172.23 ± 65.89	166.43 ± 64.03	185.84 ± 69.81	0.294
SAT volume, cm^3^, mean ± SD	447.96 ± 221.35	410.23 ± 173.79	536.63 ± 292.01	0.083
Postoperative SF-12				
MCS, mean ± SD	69.90 ± 23.68	71.40 ± 23.96	66.36 ± 23.21	0.425
PCS, mean ± SD	76.56 ± 15.52	78.72 ± 13.40	71.48 ± 19.04	0.133
Postoperative KSS				
KSC, mean ± SD	87.12 ± 6.22	88.21 ± 5.80	84.55 ± 6.55	0.038
KSF, mean ± SD	77.60 ± 12.16	83.81 ± 5.46	63.00 ± 11.05	<0.001
Postoperative VAS, mean ± SD	0.82 ± 0.67	0.74 ± 0.61	1.00 ± 0.79	0.209

BMI, body mass index; IMAT, intermuscular adipose tissue; SAT, subcutaneous adipose tissue; KSC, American Knee Society Clinical Score; KSF, American Knee Society Functional Score; VAS, Visual Analog Scale; ROM, range of motion; FC, flexion contracture; SF-12 PCS, SF-12 Physical Component Summary; SF-12 MCS, SF-12 Mental Component Summary; HU, Hounsfield units; MQ, muscle quality.

The main preoperative difference was muscle quality, which was significantly higher in the excellent-outcome group than in the non-excellent-outcome group (36.07 ± 4.08 vs. 30.04 ± 6.12 HU, *p* < 0.001). Other CT-derived tissue parameters, including skeletal muscle volume, IMAT, and SAT, did not differ significantly. At 1 year, the excellent-outcome group had better knee-specific outcomes, particularly KSC and KSF, whereas SF-12 and VAS scores were comparable between groups.

No relevant multicollinearity was detected among the five prespecified predictors, with all VIF values below 5. In the multivariable linear regression models, MQ remained independently associated with postoperative SF-12 PCS, KSC, and KSF after adjustment for age, BMI, preoperative flexion contracture, and skeletal muscle volume. Preoperative flexion contracture was also associated with postoperative SF-12 PCS, whereas skeletal muscle volume was associated with postoperative SF-12 MCS (Table 2).

**Table 2:** Multivariable linear regression models for postoperative KSC, KSF, SF-12 MCS, and SF-12 PCS.

Variable	SF-12 PCS		SF-12 MCS		KSC		KSF	
	β	*p*-value	β	*p*-value	β	*p*-value	β	*p*-value
Age	−0.114	0.713	0.061	0.782	−0.058	0.527	0.014	0.935
Pre-FC	−0.629	0.006	−0.025	0.872	−0.080	0.228	−0.131	0.271
BMI	1.199	0.160	0.411	0.491	−0.230	0.358	0.319	0.709
Skeletal muscle volume	0.055	0.064	0.046	0.017	0.004	0.581	0.012	0.396
MQ	0.993	0.006	0.673	0.098	0.464	0.008	1.100	0.002

BMI, body mass index; KSC, American Knee Society Clinical Score; KSF, American Knee Society Functional Score; FC, flexion contracture; SF-12 PCS, SF-12 Physical Component Summary; SF-12 MCS, SF-12 Mental Component Summary; MQ, muscle quality.

**Figure 3:**
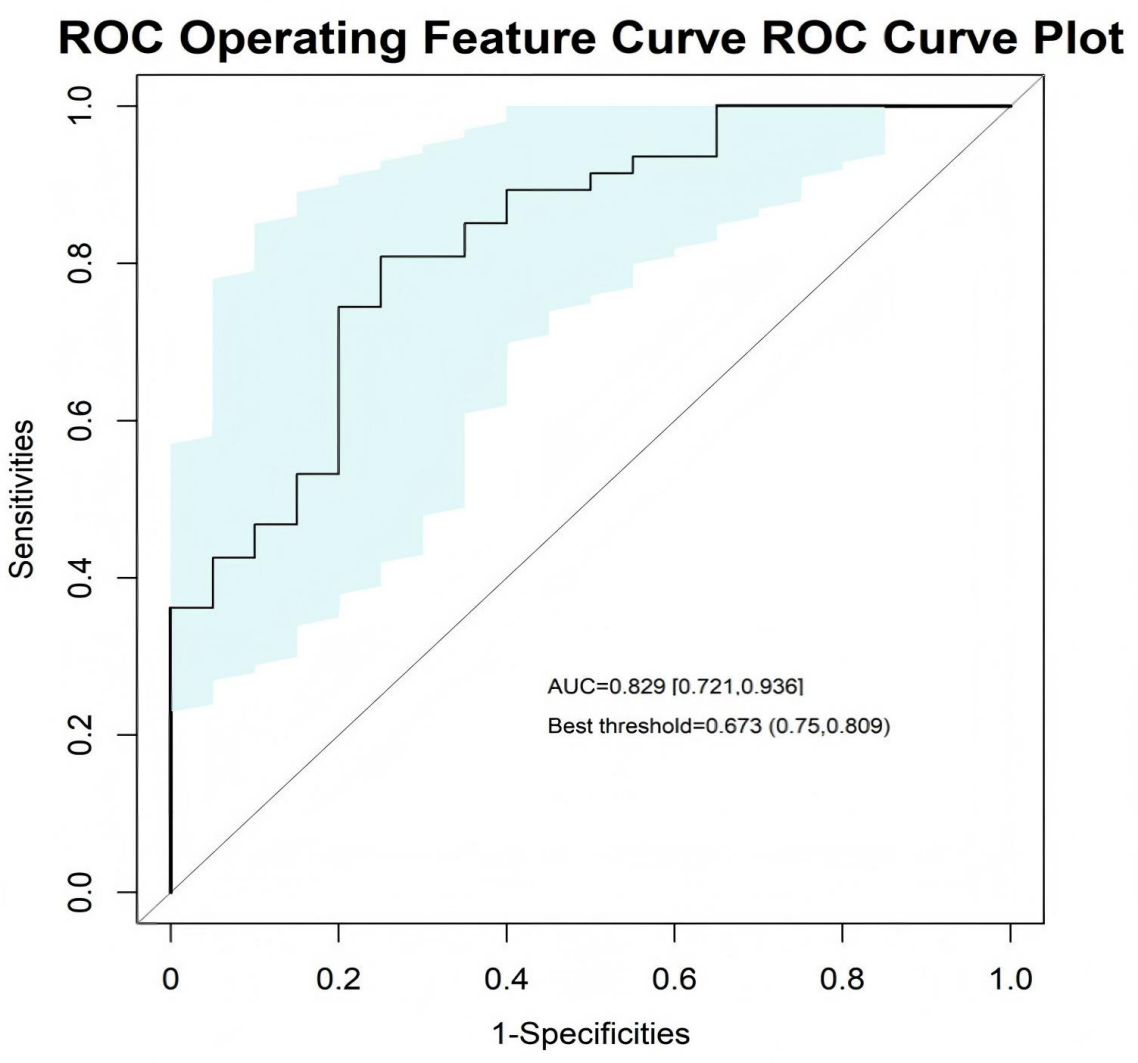
Receiver operating characteristic curve for MQ in predicting excellent postoperative outcomes. The ROC curve showed an AUC of 0.829, with a sensitivity of 80.0% and specificity of 88.9%.

In the multivariable logistic regression model, among the variables included, MQ was the only factor independently associated with achieving an excellent outcome, with each 1-HU increase associated with approximately 30% higher odds of excellent recovery (OR = 1.30, 95% CI 1.11–1.50; *p* = 0.004; Table 3). ROC analysis identified an exploratory MQ cutoff value of 31.4 HU for classification into the excellent-outcome group, with an area under the curve (AUC) of 0.829 (95% CI, 0.72–0.94), with a sensitivity of 80.0%, and specificity of 88.9% (Figure 3).

**Table 3:** Prespecified multivariable logistic regression model for excellent outcome.

Variable	OR (95% CI)	*p*-value
Age	0.969 (0.89–1.05)	0.439
BMI	1.079 (0.85–1.37)	0.526
Skeletal muscle volume	0.998 (0.99–1.03)	0.609
MQ	1.301 (1.11–1.50)	0.004
Pre-FC	0.971 (0.92–1.13)	0.272

BMI, body mass index; FC, flexion contracture; MQ, muscle quality.

The postoperative follow-up and late complications are summarized in Table 4. The mean follow-up duration was 40.6 ± 15.6 months, with a range of 13 to 60 months. There were two cases of periprosthetic fracture at 26 and 30 months after TKA, both of which were treated with open reduction and internal fixation. No aseptic loosening was observed. One case of periprosthetic joint infection occurred at 35 months after TKA and required revision surgery. Overall, the revision-free implant retention was 66 of 67 patients (98.5%) at the latest follow-up.

**Table 4:** Follow-up duration and late postoperative complications.

Variable	Overall cohort *N* = 67
Follow-up duration, months, mean ± SD	40.6 ± 15.6
Follow-up duration, months, range	13–60
Any late postoperative complication, *n* (%)	3 (4.5%)
Periprosthetic fracture, *n* (%)	2 (3.0%)
Aseptic loosening, *n* (%)	0 (0.0%)
Periprosthetic joint infection, *n* (%)	1 (1.5%)
Reoperation, *n* (%)	3 (4.5%)
Revision TKA, *n* (%)	1 (1.5%)
Revision-free implant retention at latest follow-up, *n* (%)	66 (98.5%)

Values are presented as mean ± standard deviation, range, or *n* (%). TKA, total knee arthroplasty.

### Sensitivity and subgroup analyses

The sensitivity analyses produced results that were consistent with the primary models (Supplementary Tables S1, S2). After additional adjustment for IMAT volume, muscle quality remained associated with postoperative SF-12 PCS (β = 0.756, *p* = 0.023), KSC (β = 0.336, *p* = 0.029), and KSF (β = 0.931, *p* = 0.001). The association with SF-12 MCS was attenuated and was not statistically significant (β = 0.569, *p* = 0.110). In the expanded multivariable logistic regression model with additional clinically relevant covariates, muscle quality remained independently associated with excellent postoperative outcome (OR = 1.320, 95% CI, 1.13–1.61; *p* = 0.002).

Exploratory subgroup analyses showed a generally consistent association between muscle quality and excellent postoperative outcome across clinically relevant subgroups, with no significant interaction by Arnold-Hilgartner grade (Supplementary Table S3). The hemophilia B subgroup was summarized descriptively because of the small sample size.

### Secondary analyses

The secondary matched-control analysis included 67 pairs of patients with haemophilic arthropathy and age-, sex-, and BMI-matched non-arthropathic controls (Table 5). Age and BMI remained comparable between groups (age: 38.66 ± 8.91 vs. 36.88 ± 4.92 years, *p* = 0.214; BMI: 23.72 ± 3.52 vs. 23.85 ± 2.34 kg/m^2^, *p* = 0.793). Compared with non-arthropathic controls, patients with haemophilic arthropathy had lower skeletal muscle volume (459.79 ± 165.23 vs. 548.92 ± 124.06 cm^3^, *p* = 0.005) and lower MQ (34.27 ± 5.48 vs. 42.86 ± 4.21 HU, *p* < 0.001). In contrast, SAT volume (447.96 ± 221.35 vs. 284.63 ± 82.17 cm^3^, *p* < 0.001) and IMAT volume (172.23 ± 65.89 vs. 127.95 ± 31.42 cm^3^, *p* = 0.002) were significantly higher in the hemophilia group.

**Table 5:** Comparison of CT-derived thigh muscle and adipose tissue parameters between patients with haemophilic arthropathy and non-arthropathic controls.

Variable	Haemophilic arthropathy cohort	Non-arthropathic cohort	*p*-value
	*N* = 67	*N* = 67	
Age, mean ± SD	38.66 ± 8.91	36.88 ± 4.92	0.214
BMI, mean ± SD	23.72 ± 3.52	23.85 ± 2.34	0.793
SAT volume, cm^3^, mean ± SD	447.96 ± 221.35	284.63 ± 82.17	<0.001
Skeletal muscle volume, cm^3^, mean ± SD	459.79 ± 165.23	548.92 ± 124.06	0.005
MQ (HU), mean ± SD	34.27 ± 5.48	42.86 ± 4.21	<0.001
IMAT volume, cm^3^, mean ± SD	172.23 ± 65.89	127.95 ± 31.42	0.002

BMI, body mass index; IMAT, intermuscular adipose tissue; SAT, subcutaneous adipose tissue; HU, Hounsfield units; MQ, muscle quality.

## Discussion

To our knowledge, this is among the first studies to report an imaging-derived muscle quality metric as an independent prognostic factor in haemophilic arthropathy, thereby addressing an important knowledge gap in preoperative risk stratification and patient evaluation in this population. We found that preoperative thigh muscle quality was significantly associated with postoperative outcomes after TKA in patients with hemophilia. Each 1-HU increment in muscle quality was associated with approximately 30% higher odds of achieving an excellent outcome, and an exploratory cutoff value of 31.4 HU discriminated patients with favorable outcomes.

Several biological mechanisms might explain this association. In haemophilic arthropathy, recurrent hemarthroses can induce hemosiderin deposition, chronic synovitis, inflammatory activation, cartilage damage, pain, and progressive joint deformity, thereby promoting long-term activity restriction and deterioration of periarticular muscle health (23, 24). Inflammatory mediators may contribute to skeletal muscle catabolism through activation of pathways such as NF-κB, which promotes protein degradation (25, 26). Clinically, patients with hemophilia often exhibit quadriceps wasting, weakness, and early fatigability, while persistent joint pain may induce compensatory co-contraction and reduced movement efficiency, ultimately diminishing functional muscle reserve (27, 28). Myosteatosis may represent a key intermediary mechanism linking chronic inflammation to impaired muscle performance (29, 30). Reduced CT attenuation reflects increased intramuscular lipid deposition, which can impair contractile efficiency and mitochondrial function (31). Together, these mechanisms provide a biologically plausible explanation for the association observed between lower muscle attenuation and inferior postoperative recovery in our cohort.

The secondary matched-control analysis further suggests the presence of an abnormal local muscle phenotype in haemophilic arthropathy. Compared with age-, sex-, and BMI-matched non-arthropathic controls, patients with haemophilic arthropathy showed lower thigh muscle volume and muscle quality, together with greater IMAT and SAT volumes. These findings are consistent with previous evidence showing that persons with hemophilia may have lower physical activity levels and altered body composition compared with healthy controls (32). In addition, patients with haemophilic knee arthropathy have been reported to exhibit reduced quadriceps strength, greater neuromuscular fatigue, and lower pressure pain thresholds compared with healthy peers (33). These observations provide supportive context for the impaired muscle quality observed in haemophilic arthropathy. However, this matched-control finding should be interpreted as exploratory and does not establish a causal relationship.

Prior studies across various arthroplasty populations have shown that poorer muscle quality or reduced strength is associated with worse postoperative recovery after joint replacement (34, 35). However, these data are largely derived from older degenerative populations. By focusing on patients with hemophilia and applying quantitative CT assessment, our study extends this evidence to a distinct disease context characterized by recurrent hemarthroses, chronic synovitis, and long-term functional restriction. Notably, although patients with haemophilic arthropathy had lower skeletal muscle volume than matched controls, skeletal muscle volume did not differ significantly between patients with excellent and non-excellent outcomes, nor was it independently associated with excellent postoperative outcome within the hemophilia cohort. This finding suggests that reduced muscle quantity may be a common feature of haemophilic arthropathy, whereas differences in postoperative recovery may be more closely linked to muscle composition and functional reserve. Skeletal muscle volume primarily reflects the amount of muscle tissue within the measured region, but it does not adequately account for intramuscular lipid infiltration, reduced muscle attenuation, pain-related inhibition, impaired neuromuscular activation, or force-generating capacity (28, 36). By contrast, MQ, defined as mean skeletal muscle attenuation in HU, may better capture compositional deterioration within the skeletal muscle compartment and therefore provide more clinically relevant prognostic information for postoperative functional recovery. Accordingly, our findings suggest that CT-derived muscle quality offers additional prognostic value beyond age, BMI, preoperative flexion contracture, and skeletal muscle volume.

Our findings have potential clinical implications for preoperative risk stratification and optimization. Thigh skeletal muscle quality measured on routine preoperative knee CT may reflect local muscle health and functional reserve in patients with haemophilic arthropathy. The exploratory MQ cutoff value of 31.4 HU may help identify patients with relatively poor muscle quality who are at higher risk of suboptimal functional recovery after TKA. Clinically, this threshold should not be interpreted as a definitive decision-making cutoff or used to exclude patients from surgery. Rather, patients with MQ below this value may warrant more detailed preoperative functional assessment, nutritional evaluation, and intensified perioperative rehabilitation or prehabilitation (37–39). Such information may also help surgeons and rehabilitation teams set more realistic postoperative expectations and design targeted recovery plans. However, because this cutoff was derived from a single-center retrospective cohort, prospective external validation is required before it can be incorporated into clinical decision-making algorithms.

This study should be interpreted in the light of several constraints. The cohort was modest in size and was recruited from a single tertiary referral institution, which may have reduced the external validity of the findings. Haemophilic arthropathy is a rare condition, and although we included all eligible cases over multiple years, a larger multicenter study would strengthen this evidence. Second, our retrospective design carries the inherent risks of selection bias and confounding factors. In addition, although the non-arthropathic controls were matched by age, sex, and BMI, this comparison was exploratory. The controls were retrospectively selected from imaging records rather than prospectively recruited healthy volunteers, and unmeasured factors such as physical activity level, muscle strength, and gait performance could not be fully controlled. Third, although longer-term complications and implant-related events were summarized, the primary outcome remained 1-year postoperative function. Because late events were few and follow-up duration varied, these data were descriptive and were not suitable for formal survival analysis. Fourth, muscle quality was quantified using CT attenuation from a single preoperative scan. Although CT-derived muscle attenuation is an established proxy for myosteatosis, it is not a direct measure of quadriceps strength. Direct strength measurements, such as dynamometry and objective physical performance tests, were not available; therefore, the relationship between CT-derived muscle quality and actual muscle strength could not be validated in this cohort (40).

## Conclusion

In conclusion, preoperative CT-derived thigh muscle quality is independently associated with 1-year postoperative functional outcomes after TKA in patients with haemophilic arthropathy. These findings suggest that thigh muscle quality may be a clinically relevant marker of functional reserve. However, given the retrospective design and limited sample size, prospective studies are required to confirm these observations and to determine whether interventions targeting muscle quality can improve surgical outcomes.

## Data Availability

The raw data supporting the conclusions of this article will be made available by the authors, without undue reservation.
