# Supplementary material for: Preoperative CT-derived thigh muscle quality as a predictor of postoperative outcomes after total knee arthroplasty in patients with hemophilia
Source: Front Med (Lausanne). 2026 Aug 11;13:1877333. doi: 10.3389/fmed.2026.1877333 (PMC13503179; doi:10.3389/fmed.2026.1877333)
Supplement: Supplementary file 1 [file Table_1.docx]

**Supplementary Table S1 Sensitivity analysis of multivariable linear regression models additionally including IMAT volume**

|  | **SF-12PCS** | | **SF-12MCS** | | **KSC** | | **KSF** | |
| --- | --- | --- | --- | --- | --- | --- | --- | --- |
|  | β | p-value | β | p-value | β | p-value | β | p-value |
| Age | -0.089 | 0.751 | 0.065 | 0.741 | -0.116 | 0.174 | 0.099 | 0.519 |
| Pre-FC | -0.545 | **0.012** | -0.008 | 0.954 | -0.074 | 0.239 | -0.062 | 0.584 |
| BMI | 1.359 | 0.067 | 0.493 | 0.335 | -0.001 | 0.996 | 0.208 | 0.599 |
| Skeletal muscle volume | 0.061 | 0.138 | 0.048 | **0.009** | 0.004 | 0.577 | 0.018 | 0.220 |
| MQ | 0.756 | **0.023** | 0.569 | 0.110 | 0.336 | **0.029** | 0.931 | **0.001** |
| IMAT | -0.213 | 0.065 | -0.139 | **0.003** | -0.001 | 0.610 | -0.046 | 0.191 |

BMI body mass index, KSC American Knee Society’s Clinical scores, KSF American Knee Society’s Functional scores, FC flexion contracture, SF-12PCS SF-12 Physical Component Summary scores, SF-12 MCS SF-12Mental Component Summary scores,MQ, muscle quality, IMAT, intermuscular adipose tissue.

**Supplementary Table S2 Sensitivity analysis of multivariable logistic regression models additionally including IMAT volume and clinical covariates**

| **Variable** | **OR (95% CI)** | **p-value** |
| --- | --- | --- |
| Age | 0.978(0.90,1.05) | 0.554 |
| BMI | 1.025(0.82,1.26) | 0.821 |
| Skeletal muscle | 0.996(0.99,1.01) | 0.910 |
| MQ | 1.320(1.13,1.61) | **0.002** |
| IMAT | 0.996(0.97,1.01) | 0.613 |
| Arnold-Hilgartner grade | 0.568(0.11,2.87) | 0.501 |
| Pre-ROM | 0.982(0.95,1.02) | 0.265 |
| Pre-FC | 0.962(0.92,1.02) | 0.288 |

BMI body mass index, FC flexion contracture, MQ, muscle quality, IMAT, intermuscular adipose tissue, ROM range of motion.

**Supplementary Table S3. Exploratory subgroup analyses of the association between MQ and excellent postoperative outcome after adjustment for age and BMI**

| **Subgroup variable** | **n (%)** | **OR** | **95% CI** | **p-value** | **p for interaction** |
| --- | --- | --- | --- | --- | --- |
| Overall | 67 (100.0) | 1.30 | 1.11-1.52 | 0.001 | — |
| Arnold-Hilgartner grade |  |  |  |  | 0.708 |
| Grade IV | 24 (35.8) | 1.25 | 1.00-1.58 | 0.050 |  |
| Grade V | 43 (64.2) | 1.33 | 1.08-1.65 | 0.008 |  |
| Haemophilia type |  |  |  |  | — |
| Type A | 60 (89.6) | 1.29 | 1.10-1.50 | 0.002 |  |
| Type B | 7 (10.4) | Not estimable | — | — |  |

Covariate adjustment: Age, BMI. Because the number of patients with haemophilia B was small, the type B-specific estimate was not considered statistically stable and was summarized descriptively. MQ, muscle quality; HU, Hounsfield units; OR, odds ratio; CI, confidence interval; BMI, body mass index.
